# Successful nonsurgical management of Oehler’s type III dens invaginatus in maxillary lateral incisor: A case report as per CARE guidelines

**DOI:** 10.1097/MD.0000000000042725

**Published:** 2025-08-01

**Authors:** Anshul Sachdeva, Gurdeep Singh Gill, Adel Al Obied, Suraj Arora, Ali Y. Alsaeed, Waled Abdulmalek Alanesi, Gotam Das

**Affiliations:** a Department of Pedodontics and Preventive Dentistry, D.J College of Dental Sciences and Research, Meerut, UP, India; b Department of Conservative Dentistry and Endodontics, Jan Nayak Choudhary Devilal (JCD) Dental College, Sirsa, India; c Department of Restorative Dental Sciences, College of Dentistry, King Khalid University, Abha, Saudi Arabia; d Department of Operative Dentistry, Faculty of Dentistry, University of Science and Technology, Inmaa City, Aden, Yemen; e Department of Prosthodontics, College of Dentistry, King Khalid University, Abha, Saudi Arabia.

**Keywords:** apexification, apical periodontitis, dens invaginatus, endodontic treatment, MTA, type III dens invaginatus

## Abstract

**Rationale:**

Dens invaginatus (DI) is a rare condition with a prevalence of only 0.3% to 10% mainly affecting the maxillary lateral incisors. In the present case, we describe management of the rarest variant of DI, that is, Oehler type III DI.

**Patient concerns:**

Patient complained of severe pain in upper left front teeth since 1 month which aggravated on taking hot liquids. Patient had visited a general dentist who did emergency access opening on #9 and #10 and referred the patient.

**Diagnoses:**

Oehler type III DI with apical periodontitis w.r.t.#10 (maxillary left lateral incisor).

**Interventions:**

Nonsurgical root canal treatment of the main canal and apexification of pseudo canal with mineral trioxide aggregate.

**Outcomes:**

At 6 months follow-up, the patient was asymptomatic, and the clinical evaluation revealed no sensitivity to percussion or palpation on tooth #10 and mobility within normal limits. The radiograph examination displayed significant periapical healing.

**Lessons:**

The unusual morphological appearance of the crown in the maxillary lateral incisor or the radiographic appearance should alert the dentist to the possibility of DI. Knowledge of tooth anatomy and its variations, correct diagnosis, and use of magnification and advanced materials like mineral trioxide aggregate can help in managing rare variations.

## 1. Introduction

A successful endodontic treatment hinges on the knowledge of variations in root canal anatomy and morphology. One such variation, though rare, detected in maxillary lateral incisors is dens invaginatus (DI). It is thought to arise because of an invagination into the surface of the tooth crown before calcification has occurred.^[1]^ This is also referred to as dens in dente, invaginated odontome, dilated gestant odontome, dilated composite odontome, tooth inclusion, dentoid in dental literature.^[2]^ The actual cause is still unclear, but it is thought to occur because of genetic and environmental factors.^[2–4]^ Oehlers classified the DI into coronal DI and radicular DI based on the origin of invagination.^[5]^ Coronal DI was further classified into 3 types according to the depth of invagination observed radiographically. Type I is enamel-lined invagination confined to the coronal part of the tooth; Type II is extension of the invagination beyond the cementoenamel junction ending as a blind sac. Type III includes the permeation of the root by the invagination to form an additional canal opening on the lateral side of the root.^[5]^ This additional canal has been termed a pseudo canal by Gonsalves et al.^[6]^ This pseudo root canal is generally regarded as the usual site of origin of infection.^[7]^

The prevalence of DI ranges from 0.3 to 10% with type I being most common and type III least common. Maxillary lateral incisor is the most affected tooth,^[2–4,8]^ but cases with maxillary central incisor, maxillary canine, and mandibular central incisor have been reported.^[9–11]^ The DI is frequently associated with morphological defects like dilated or peg-shaped grooving of the palatal enamel, conical or barrel-shaped crown, or dilated morphology, or has a bifid exaggerated cingulum or a deep foramen cecum, which makes the tooth susceptible to caries and subsequent pulpitis.^[12]^ The endodontic treatment is further complicated by difficult instrumentation access, presence of dips, concavities, intracanal communications, inaccessible fins, apical ramifications, and open apices.^[11]^ DI is commonly detected as an incidental radiographic finding if the patient is not symptomatic. In case of atypical anatomy of crown it is recommended to perform radiographic examination. The dens appears as a radiolucent cavity lined by radiopaque enamel in the crown or extending to the root. It may be associated with periapical or lateral radiolucency. But radiographs, being 2-dimensional, are insufficient to fully disclose the complex anatomy of DI, and inherent problems like noise, geometric distortion, and overlapping of structures can affect the diagnostic accuracy. Despite the disadvantages of radiography, it is still readily available at every dental clinic and affordable.^[13]^ Cone beam computed tomography (CBCT) can provide 3-dimensional imaging of the lesion, its association with pulp space, and define the type of DI and differentiate it from radicular groove, and help in treatment planning.^[12,13]^ But radiation dose, availability, and cost may be concern for some patients. The management of DI aims at either prevention of pulpal pathology or to help treat an affected pulp with or without apical periodontitis.

The present case report describes the conservative management of a rare type III DI in a maxillary lateral incisor.

## 2. Case report

### 2.1. Patient information

An Indian origin 15-year-old male patient was referred to the Department of Endodontics by a general dentist. Patient complained of severe pain in upper left front teeth since 1 month. Pain was severe and aggravated on taking hot liquids. Patient had visited the referring general dentist who had suggested root canal treatment and did emergency access opening on #9 and#10 and referred the patient. Patient was taking Ketorolac 10 mg (Ketorol DT, DrReddy’s) for relief of pain. Patient had no relevant medical history.

### 2.2. Clinical findings, timeline, and diagnostic assessment

**Figure 1.**
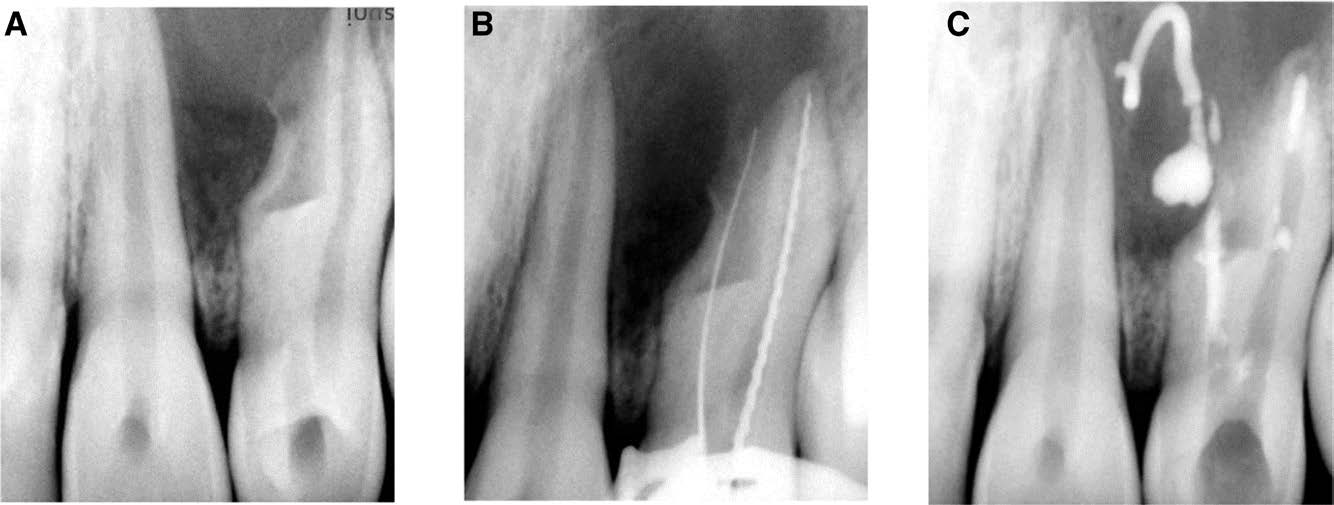
(A) Periapical radiograph of tooth #10 displaying DI Oehler type III, along with wider dimension mesiodistally compared to tooth #9, complex internal anatomy, and periapical radiolucency along with previous access cavities in teeth #9, #10. (B) Periapical radiograph of tooth #10 to confirm the working of the regular root canal on the distal side and the pseudo canal on the mesial side. (C) Inter-appointment application of calcium hydroxide paste in the canals with some overflow on the mesial side of tooth #10. DI = dens invaginatus.

There was no extraoral swelling present. On intraoral examination, teeth #9 and #10 had crowns with normal morphological appearance on buccal side while on lingual side access openings were visible. Both teeth were tender on percussion and grade I mobile. Teeth did not respond to vitality testing with electric pulp tester (Digitest; Parkell Inc, Brentwood, New Jersey) and cold test (Endo Ice 1,1,1,2 tetrafluoroethane Coltene Whaledent Inc, Ohio). Radiographic examination (Fig. 1A) revealed unusual configuration of root canals with respect to tooth #10, with one completely formed main root canal and an invagination laterally, on the mesial side (pseudo canal). This pseudo canal had an open apex with no evident communication with main canal, and the tooth was associated with a periapical radiolucency. This radiographic appearance confirmed to the Oehlers type III DI in relation to tooth #10.

Based on clinical and radiographic findings, a diagnosis of previously initiated therapy with symptomatic apical periodontitis with respect to teeth # 9 and #10 was made. The patient and the parents were informed about the diagnosis. All treatment options were discussed, which included conservative root canal treatment of the main canal with apexification of the pseudo canal, pulp revascularization, surgical treatment if conservative management fails, and extraction as a last resort. The parents gave consent for conservative management of tooth #10 with conventional root canal treatment and apexification.

### 2.3. Therapeutic intervention

Under local anesthesia (1.8 mL of 2% lignocaine containing 1:200,000 adrenaline [Lignocad-Adr, Cadila Pharmaceuticals Ltd, Samba, Jammu and Kashmir, India] and rubber dam isolation), access cavity (prepared by referring dentist) was modified for unimpeded access to main canal and exposed the pseudo canal by removing the invagination covering it. Rubber dam was held in place with wedges (Coltene Whaledent Inc) so that clamps do not superimpose on the crucial coronal part anatomy during radiographs.

Working length of main canal and pseudo canal was determined using apex locator (Root Zx, J Morita, Tokyo, Japan) and confirmed using periapical radiograph (Fig. 1B). Biomechanical preparation of main canal was done with step back technique up to size # 40 stainless steel K file (Mani Inc., Utsunomiya, Tochigi, Japan). The large pseudo canal was prepared after enlarging the opening with Gates Glidden drills (#4, #5) and using H files (Mani Inc.) circumferentially. Throughout biomechanical preparation, irrigation was done with 2.5% sodium hypochlorite activated by ultrasonics, and after completion, 1 mL of 17% EDTA was used for smear layer removal. Calcium hydroxide paste (Metapex, Meta Biomed Co. Ltd, Cheongju, Korea) was placed as an inter-appointment medicament and sealed with Cavit (3M ESPE AG, Seefeld, Germany) for 1 week. Some of calcium hydroxide overflowed out of the pseudo canal (Fig. 1C). Patient was informed about the mishap and told to expect some mild discomfort because of that and return to office in case of severe pain or swelling. Patient was recalled after 1 week.

**Figure 2.**
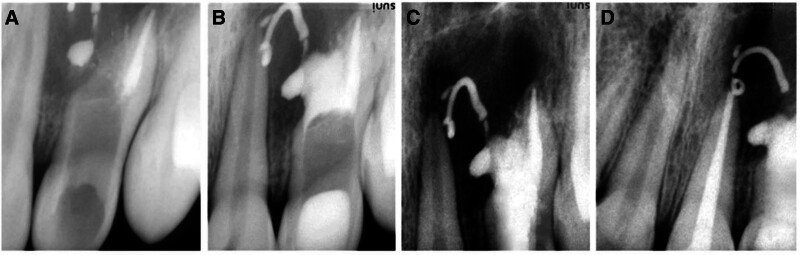
(A) Obturation of regular root canal and removal of intertwining invaginated tissue between the root canal and the pseudo canal in tooth #10. (B) Apexification of the pseudo canal by creating a 4 to 5 mm apical plug using mineral trioxide aggregate in tooth #10. (C) Middle and coronal third of the canal obturated with warm vertical condensation technique using the Obtura III Max in tooth #10. (D) Periapical radiograph of teeth #9, #10 at 6 months recall displaying significant periapical healing.

Patient remained asymptomatic and in the second appointment the root canals were ultrasonically irrigated, calcium hydroxide was removed, and the intertwining invaginated tissue between the pulp canal and the pseudo canal at middle 3rd of the root canal was removed completely using the Gates Glidden drills sizes #5, #6 (Fig. 2A). Both canals were irrigated with 2.5% sodium and canals were dried with paper points. Main pulp canal was obturated with lateral condensation technique (Fig. 2A). A 4 to 5 mm mineral trioxide aggregate (ProRoot MTA, Maillefer, Dentsply, Baillaigues, Switzerland) apical plug was created for the apexification of the open pseudo canal (Fig. 2B) and covered with wet cotton. After temporary restoration, the patient was recalled next day, and pseudo canal was obturated with warm vertical condensation technique using the Obtura III Max (Obtura Spartan Endodontics, Algonquin) (Fig. 2C). The access cavity was restored with composite resin (Solitaire 2, Heraeus Kulzer, Wehrheim, Germany). Endodontic treatment of adjacent tooth #9 was completed in subsequent visits.

### 2.4. Follow-up and outcomes

Patient remained asymptomatic after 6 months of follow-up. Clinical examination revealed no tenderness to percussion, palpation, or mobility within normal limits. Radiographic examination showed signs of periapical healing. The calcium hydroxide in the periapical area was also resorbing albeit slowly (Fig. 2D). Patient was contacted for further follow-ups at regular intervals, but has expressed his inability due to relocation to another city for further education.

## 3. Discussion

The management of DI includes preventive sealing/filling of the invagination, nonsurgical root canal treatment, apexification or regenerative endodontic procedures, periradicular surgery, intentional replantation, or extraction. The present case had all the typical challenges associated with management of type III DI, like complex root canal anatomy with invagination pressing on the coronal and middle part of main canal, presence of pseudo canal with open apex, pulpal necrosis, and presence of apical periodontitis. The various treatment options for endodontic management of such cases are early recognition and diagnosis and conservative nonsurgical endodontic treatment, where only invagination is treated and tooth vitality is preserved,^[14–17]^ nonsurgical endodontic treatment of both main canal and pseudo canal,^[9,18–24]^ combination of nonsurgical and surgical endodontic procedures,^[10,25–29]^ revascularization^[29–31]^ and even replantation.^[32]^ In virtually all cases of DI, it is recommended to approach the invagination, regardless of the pulp condition, either to prevent pulp pathology or to help treat a tooth with necrotic pulp with or without apical periodontitis.^[12]^

The management of the case starts with early recognition of the condition. In a review, Zhu et al (33) have outlined the clinical presentations that can alert clinicians about the possible presence of DI. These include the presence of a palatal pit or groove (palatal pit or groove always is the entrance of the invagination), barrel or cone-shaped teeth, dilated/enlarged crown (compared with contralateral or adjacent teeth), microdontic teeth (rare), or talon cusp or dens evaginatus, or presence of a labial groove.^[33]^ As the present case was a referral, we were not able to see the initial morphology, and main root canal was already accessed by the referring dentist. But periapical radiolucency involving the main canal was visible on radiograph, indicating involvement of main canal. Though radiographically no visible communication was present between pseudo canal and pulp chamber, indirect communication through the defects that might be present in the enamel and dentin surrounding the invagination could have infected the pulp in the present case.^[3,22]^ So, it was decided to manage the tooth with nonsurgical endodontic treatment of both main canal and pseudo canal, as successfully done in previous case reports.^[9–11,18–24]^ Due to economic reasons, CBCT of the area could not be conducted, which would have provided better assessment of main canal. If main canal was not involved, a conservative treatment where only invagination is treated, thus preserving the tooth vitality, may be the best course of action in this case. Case reports with successful outcomes by treating only the invagination have been published in the literature.^[14–18]^

The preparation of the access cavity is challenging technically in these cases because of the location of the pulp chamber and the invagination. Dental operating microscope plays an important role in conservative access preparation. Complete disinfection of the root canal is necessary for a predictable outcome; this can be achieved by unimpeded access to instrumentation and irrigants. In the present case, the invaginated tissue was occupying most of the coronal and middle part of root canal, so we removed the complete invagination with Gates Glidden drills to improve accessibility, as done in a previous case report.^[34]^ The sodium hypochlorite irrigant was further activated ultrasonically to improve its effectiveness and reach to clean both canals effectively. Calcium hydroxide paste was used as intracanal medicament as it helps in obtaining predictable debridement and decontamination of infected root canals.^[35]^

As the pseudo canal had an open apex, MTA was used for apexification because of its sealing ability, biocompatibility, and ability to form a hard tissue barrier, similar to other case reports.^[30,36]^ Though in the case of very short roots and thin walls, revascularization has also been reported with a successful outcome.^[29,30]^ In our case main root canal was fully formed, so we decided to close the invagination with an MTA barrier.

Though CBCT could have provided more information about the complexity of DI, which could help in its management, the unavailability of CBCT at the treatment Centre and limited affordability of the patient precluded its use in the present case. A longer follow-up period could provide further insights about the outcome of treatment. Newer technologies like guided endodontics and computer-aided dynamic navigation may be used for planning and more accurate management of dens.^[37]^

## 4. Conclusion

Type III DI is a rare condition mainly affecting maxillary lateral incisors. The unusual morphological appearance of the crown should alert the dentist about the possibility of DI, and findings should be confirmed with diagnostic radiographs and CBCT, if available. The management should aim at conservative treatment and complete removal of irritants from the canal. Use of newer technologies like Dynamic navigation and guided endodontics, and contemporary materials like MTA can help in achieving a successful, predictable outcome in most cases.

## Author contributions

**Conceptualization:** Anshul Sachdeva.

**Data curation:** Gurdeep Singh Gill, Ali Y. Alsaeed.

**Funding acquisition:** Suraj Arora.

**Investigation:** Anshul Sachdeva.

**Methodology:** Gotam Das.

**Project administration:** Adel Al Obied.

**Software:** Gotam Das.

**Validation:** Waled Abdulmalek Alanesi.

**Visualization:** Gurdeep Singh Gill, Ali Y. Alsaeed.

**Writing – original draft:** Suraj Arora, Gotam Das.

**Writing – review & editing:** Waled Abdulmalek Alanesi.
